# Epidemics of Kentucky and Tennessee

**Published:** 1851-07

**Authors:** 


### EPIDEMICS OF KENTUCKY AND TENNESSEE.

Our readers will remark that Dr. Sutton, of Georgetown, has been placed, by the American Medical Association, at the head of the committee on the *Epidemics of Kentucky and Tennessee*, and will agree with us that a better selection could not have been made. Dr. Sutton is a gentleman of great industry, and excellent judgment, and we shall look with much interest for his report on this important subject. Y.

